# Economic Value of Dengue Vaccine in Thailand

**DOI:** 10.4269/ajtmh.2011.10-0624

**Published:** 2011-05-05

**Authors:** Bruce Y. Lee, Diana L. Connor, Sarah B. Kitchen, Kristina M. Bacon, Mirat Shah, Shawn T. Brown, Rachel R. Bailey, Yongjua Laosiritaworn, Donald S. Burke, Derek A. T. Cummings

**Affiliations:** University of Pittsburgh School of Medicine, Pittsburgh, Pennsylvania; University of Pittsburgh Graduate School of Public Health, Pittsburgh, Pennsylvania; Pittsburgh Supercomputing Center, Pittsburgh, Pennsylvania; Bloomberg School of Public Health, Johns Hopkins University, Baltimore, Maryland

## Abstract

With several candidate dengue vaccines under development, this is an important time to help stakeholders (e.g., policy makers, scientists, clinicians, and manufacturers) better understand the potential economic value (cost-effectiveness) of a dengue vaccine, especially while vaccine characteristics and strategies might be readily altered. We developed a decision analytic Markov simulation model to evaluate the potential health and economic value of administering a dengue vaccine to an individual (≤ 1 year of age) in Thailand from the societal perspective. Sensitivity analyses evaluated the effects of ranging various vaccine (e.g., cost, efficacy, side effect), epidemiological (dengue risk), and disease (treatment-seeking behavior) characteristics. A ≥ 50% efficacious vaccine was highly cost-effective [< 1× per capita gross domestic product (GDP) ($4,289)] up to a total vaccination cost of $60 and cost-effective [< 3× per capita GDP ($12,868)] up to a total vaccination cost of $200. When the total vaccine series was $1.50, many scenarios were cost saving.

## Introduction

As a human dengue vaccine moves closer to the market, understanding its potential economic value can help guide manufacturers, policy makers, public health officials, clinicians, and other decision makers during the vaccine's final stages of development and potential implementation. A dengue vaccine would fill a substantial need, as dengue can result in substantial morbidity, healthcare resource use, and productivity losses and affects an estimated 70,000,000 to 500,000,000 people worldwide annually, with ~3.6 billion people at risk for infection.^1^ Limitations of vector control strategies have allowed dengue to remain endemic in countries such as Thailand, where it is one of the most common causes of child hospitalization.^2^–^4^ Moreover, the geographic range of dengue vectors, *Aedes aegypti* and *Aedes albopictus*, continues to expand into new regions throughout the world, including the southern United States.^3^,^5^

The substantial, potentially growing morbidity and mortality of dengue have motivated development of a dengue vaccine. Four closely related dengue serotypes exist, all of which currently circulate in Thailand.^6^,^7^ Although infection grants lifelong immunity against the infecting serotype, prior infection results in an increased likelihood of dengue hemorrhagic fever (DHF), during subsequent (i.e., second) exposure to a different serotype.^8^–^10^ An ideal vaccine would therefore simultaneously induce complete immunity to all viral serotypes.^11^,^12^ A number of vaccines are currently under various stages of clinical and pre-clinical development. A tetravalent ChimeriVax dengue vaccine (manufactured by Sanofi Pasteur, Lyon cedex, France) has completed phase IIb clinical trials in Thailand, which is the first trial to provide some indication of the potential for clinical benefit by actively immunizing against dengue, and is now undergoing phase III evaluation.^13^,^14^ GlaxoSmithKline (GSK) and Walter Reed Army Institute of Research (WRAIR) are developing a live-attenuated vaccine currently in phase II clinical trials^15^ and the National Institute of Allergy and Infectious Diseases (NIAID) has an infectious clone vaccine currently in phase I clinical trials.^12^ Several vaccine candidates in preclinical development include a whole purified inactivated virus vaccine (developed by WRAIR), a replication-incompetent virus vaccine (developed by Novartis), viral protein subcomponent vaccines (developed by Hawaii Biotechnology and Pedro Kourí Institute of Tropical Medicine/Genetic Engineering and Biotechnology Center), a DNA vaccine (developed by the U.S. Navy), and virus vector (e.g., adenovirus and measles virus) vaccines (developed by GenPhar Inc. and the Centre National de la Recherche Scientifique).^12^

Answering key questions about a vaccine's economic value before licensure, when vaccine characteristics and strategies can potentially be altered, can facilitate its chances of success and guide its implementation.^16^ We constructed a computational model to evaluate the economic value of vaccinating individuals in Thailand. Prior economic studies have examined the use of vector control programs.^4^,^17^,^18^ A previous analysis by Shepard and others^2^ (performed before phase II trials were completed and more recent data were available) used a deterministic model to show the cost-effectiveness of a pediatric vaccine in Southeast Asia.^13^ Questions about how the value of a dengue vaccine may vary by vaccine cost, vaccine efficacy, and dengue risk can be addressed more accurately with the most recent data provided by the phase II trials, and new questions such as effects of various treatment-seeking behavior can be assessed. Our aim is to extend Shepard's study by incorporating more recent data (e.g., a 3-dose versus a 2-dose vaccine, clinical and vaccine data since 2003, etc.), stochasticity (i.e., using distributions instead of point estimates), a more extensive representation of the disease (e.g., different exposure states such as primary or secondary infection, and age-specific disease outcomes within those exposure states, etc.), and dengue data specific to Thailand. Sensitivity analyses explored the effects of varied key parameters such as vaccine cost (to help establish price points), vaccine efficacy (to identify vaccine efficacy targets), and risk of infection (to evaluate different target populations).

## Methods

### Model structure.

**Figure 1.**
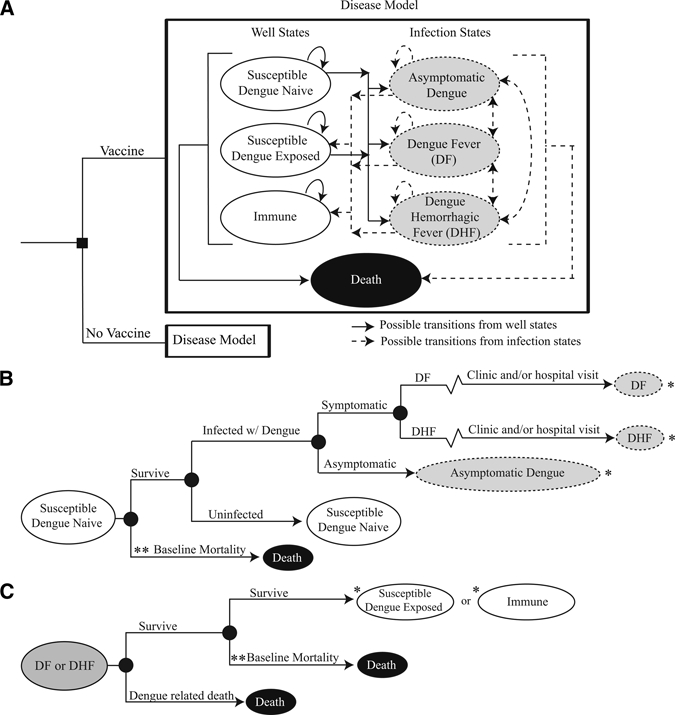
General model structure: (**A**) Disease Model, (**B**) Disease Model Subtree for paths of an individual who begins in the Susceptible Dengue Naive state. (**C**) Disease Model Subtree for paths of an individual after being infected with either form of symptomatic dengue; dengue fever (DF) or dengue hemorrhagic fever (DHF). *Once a “Susceptible Dengue Naive” individual gets an infection with symptomatic or asymptomatic dengue, after they recover, they can only go to the “Susceptible Dengue Exposed” state to begin new probabilities of infection. Once an individual has experienced 2 infections, they go to the “Immune” state. **Refers to mortality resulting from causes unrelated to dengue (based on Thailand-specific life expectancy and mortality tables).

Using TreeAge Pro 2010 (TreeAge Software, Williamstown, MA), we developed a decision analytic Markov simulation model to evaluate the potential health and economic value of administering a dengue vaccine to a dengue-naive individual (≤ 1 year of age) in Thailand from the societal perspective (Figure 1). Following the initial vaccination decision node, the individual proceeds into the Markov portion of the model, consisting of the following seven Markov states:

• **Well/susceptible (dengue-naive):** In this state, the individual is uninfected for the duration of the cycle (i.e., 1 year) and has never been previously infected. (Infants < 1 year of age are a unique group in that maternal antibody may protect them in the first months of life, but as antibody titers decrease they lose their protective ability and can enhance dengue infection, thereby predisposing infants to develop DHF/dengue shock syndrome [DSS] with their first dengue infection.^19^)
• **Well/susceptible (dengue-exposed):** In this state, the individual is uninfected for the duration of the cycle (i.e., 1 year) and was previously infected once (i.e., acquired immunity to one serotype).
• **Immune:** An individual is immune to further infections, as a result of having had at least two natural dengue infections.
• **Asymptomatic dengue infection:** The individual experiences a dengue infection but remains asymptomatic throughout the infection. An asymptomatic infection is defined in the model according to the classification used by Burke and others^9^ as a lack of symptoms or minimal symptoms as measured by an absence from school of < 1 day. Infection results in acquisition of immunity to the infecting serotype.
• **Dengue fever (DF):** The individual experiences symptomatic dengue fever (DF), which can consist of fever, headache, nausea, and muscle and/or joint pain.
• **Dengue Hemorrhagic Fever/Dengue Shock Syndrome (DHF/DSS):** An individual experiences a more severe form of disease, including vascular leakage, hemorrhagic manifestations, thrombocytopenia, and fever.
• **Death:** An individual reaches this state from death either attributed to dengue (DF or DHF/DSS) or an unrelated cause of mortality (based on life expectancy/mortality tables from Thailand).^20^ Death is an absorptive state; once the individual reaches this state, the individual's simulation ends.

During each cycle (i.e., 1 year) an individual had probabilities of staying in the same state or transitioning into another state. The arrows in Figure 1A show the possible movements among states. Figure 1B displays the potential paths that an individual could take upon entering each state when beginning at the susceptible dengue naive state; the likelihood that an individual traveled down each path was determined by flat probability values and distributions from the literature. Table 1 lists these probabilities. Figure 1C indicates how an individual moves from a state of symptomatic dengue (DF or DHF) to begin at another state. When infected, an individual in the Well/Susceptible state could have asymptomatic dengue, develop DF or develop DHF/DSS. Individuals who had previously been infected had an increased probability of progressing to more severe manifestations of disease, i.e., DHF/DSS than those who were dengue-naive. Some literature (from Cuba, because Thailand data is not available) suggests that children also carry a higher probability of having clinical manifestations of dengue infections than adults.^21^,^29^ A ratio of 3.457:1 (cases of DHF/DSS in children to cases of DHF/DSS in adults) found by Guzman and others^21^ was used to convert adult to child probabilities.

Vaccinated individuals had a decreased chance of acquiring a dengue infection based on vaccine efficacy, i.e., vaccinated infection risk = uninfected infection risk * (1-vaccine efficacy). This vaccinated population had probabilities of developing side effects to the vaccine similar to those associated with other flavivirus vaccines (yellow fever and Japanese encephalitis [JE]). Common minor side effects were localized pain, swelling, fever, and aches. Recent phase I trials of a dengue vaccine^30^ and studies of another flavivirus vaccine (ChimeriVax-JE vaccine) suggest that the risk of a major side effect may be exceedingly low.^31^–^33^ However, to be conservative about the benefits of the vaccine in our model, vaccinated individuals could develop vaccine-induced DHF, which had a chance of leading to death, at a probability that was derived from another flavivirus (yellow fever virus) vaccine's documented major side effect (yellow fever vaccine-associated viscerotropic disease, YEL-AVD).^24^ The cost per each minor side effect event was equivalent to the purchase of a 10-pack over the counter pain reliever (Laosiritaworn Y, personal communication). Major side effects of vaccination led to death 50% of the time; otherwise, it led to a DHF disability-adjusted life-year (DALY) decrement. The baseline distribution of major and minor side effect probabilities were based on yellow fever and JE vaccine safety reports, and recently released data from dengue vaccine phase II trials regarding adverse event frequency.^13^,^24^,^25^

Each simulation run sent 1,000 individuals through the model 1,000 times equating to one million realizations. Each individual cycled through the model until they entered the Death state. For each simulation run, the following formula calculated the incremental cost-effectiveness ratio (ICER), or the cost per DALY averted, of vaccination:
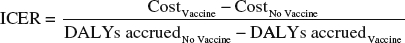


As per the World Health Organization (WHO) convention, the cost-effectiveness threshold was based on Thailand's gross domestic product (GDP) per capita ($4,289).^34^,^35^ Vaccination was considered highly cost-effective when the ICER was less than the GDP per capita ($4,289 per DALY averted), cost-effective when the ICER was between one and three times the GDP per capita ($4,289–$12,868 per DALY averted), and not cost-effective when the ICER exceeded three times the GDP per capita (> $12,868 per DALY averted).^36^

The model also recorded the simulated number of total dengue (asymptomatic and symptomatic dengue fever and DHF/DSS) cases averted by vaccination. The cost per avoided dengue and DHF/DSS case was calculated by dividing the incremental cost (the cost difference between vaccination and no vaccination) by the number of cases that vaccination averted.

### Data inputs.

Table 1 lists probabilities, costs, disability weights, and time missed from work or school with their corresponding sources. Thailand-specific life expectancies and crude mortality rates as well as disability weights for DF and DHF/DSS were obtained from the WHO.^20^,^27^ DALYs accrued from two sources: symptomatic dengue illness and vaccine side effects that caused years lost caused by disability (YLD); and death, which resulted in years of life lost (YLL) from the remaining life expectancy. The following formula calculated DALYs:
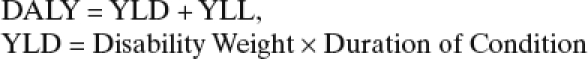


A generally accepted discount rate of 3% updated costs to 2010 United States dollar (USD) and current exchange rates converted results in USD and Thai Baht (THB), with a conversion rate of $1US = 31.1632THB.^37^,^38^

### Sensitivity analyses.

Sensitivity analyses varied the values of key parameters such as vaccination cost (range: $1.50 to $800 total for 3 doses), vaccine efficacy (range: 50–95%), dengue infection risk (baseline: 9%; range: 5–15%), ratios of DHF in children and adults (1:1 and 5:1), primary and secondary symptomatic infections resulting in DHF (15% and 75%, respectively, and 10% and 50%, respectively), secondary asymptomatic rates (75% and 90%) and treatment-seeking behavior (i.e., probability of visiting a clinic, hospital, both or neither).^2^,^39^

Different experiments explored the effects of using three different treatment-seeking scenarios. The treatment-seeking behavior affected the health care resources used and therefore health care costs (Laosiritaworn Y, personal communication).^9^

### Scenario 1 (high estimate of treatment-seeking behavior):

DF

• Probability of visiting a clinic = 50%
• Probability of visiting a hospital (with or without prior clinic visit) = 5%

DHF/DSS

• Probability of visiting a clinic = 75%
• Probability of visiting a hospital (with or without prior clinic visit) = 75%

### Scenario 2 (middle estimate of treatment-seeking behavior):

DF

• Probability of visiting a clinic = 35%
• Probability of visiting a hospital (with or without prior clinic visit) = 5%

DHF/ DSS

• Probability of visiting a clinic = 60%
• Probability of visiting a hospital (with or without prior clinic visit) = 60%

### Scenario 3 (low estimate of treatment-seeking behavior):

DF

• Probability of visiting a clinic = 25%
• Probability of visiting a hospital (with or without prior clinic visit) = 5%

DHF/ DSS

• Probability of visiting a clinic = 50%
• Probability of visiting a hospital (with or without prior clinic visit) = 50%

## Results

### Overall impact.

Results showed vaccination to be cost-effective, and in many cases highly cost-effective across a wide range of scenarios until the vaccination cost was greater than $200. In fact, with a vaccination price point of $1.50 for the vaccination series, administration of the vaccine was actually cost saving. At the baseline incidence rate of infection (9%), which includes symptomatic and asymptomatic forms, vaccinating only became not cost-effective (i.e., ICER > 12,868/DALY averted), when the vaccination cost reached $300 and was accompanied by a vaccination efficacy of 50% or lower, and at a vaccination cost of $500 with a vaccine efficacy of 75% or lower. Additionally, the cost of averting a case of dengue was often less than $100 when vaccination costs were under $60. Varying the likelihood of minor and major side effects of vaccination, the ratio of DHF in children versus adults and of treatment-seeking behaviors had minimal impact on model results.

### Cost-effectiveness of vaccination.

**Figure 2.**
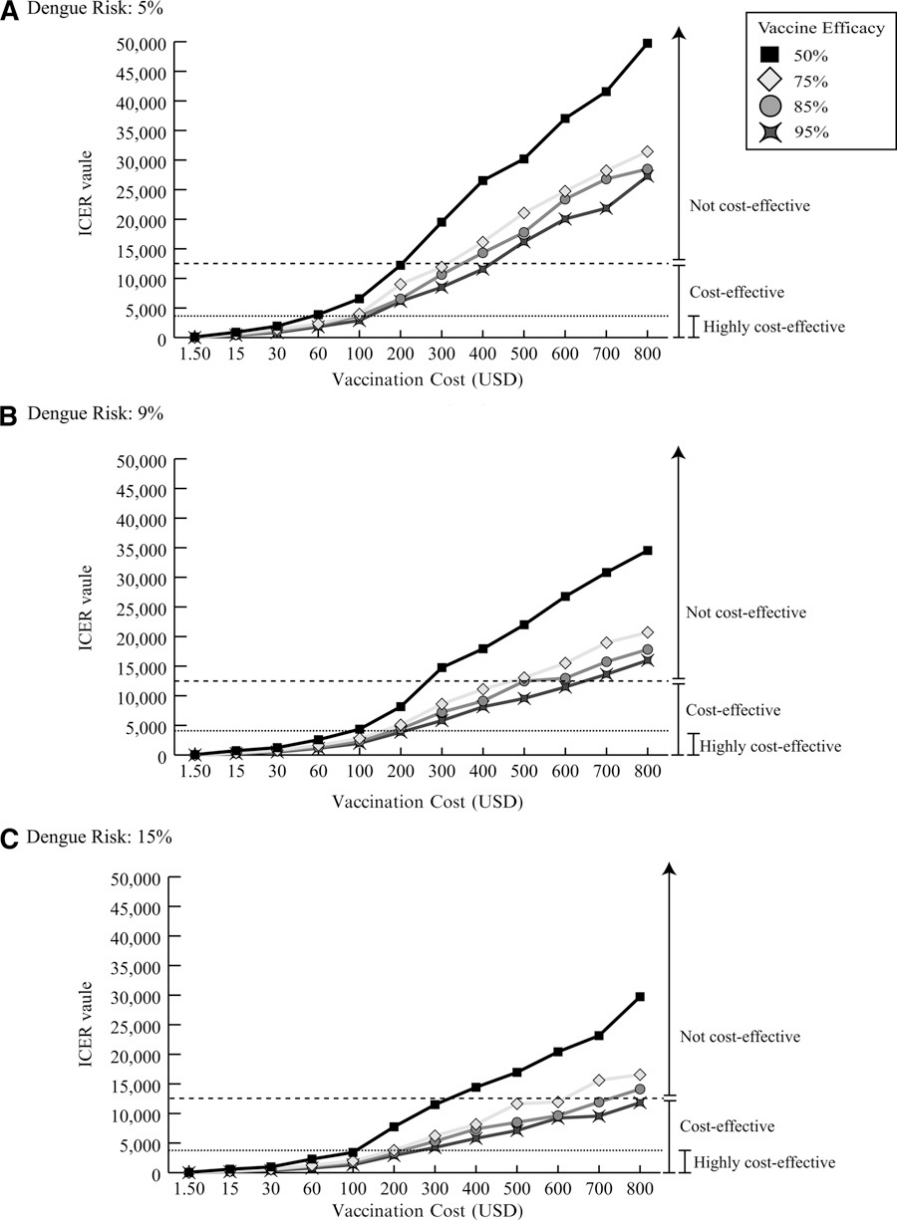
Incremental cost-effectiveness ratio (ICER) of dengue vaccination in U.S. dollars. Strategies are highly cost-effective at and ICER of < 4,289 (dotted line) and remain cost-effective until 12,868 (dashed line), where it becomes not cost-effective. (**A**) Dengue risk 5%, (**B**) dengue risk 9%, and (**C**) dengue risk %15

Figure 2 shows the effect of infection risk (including both symptomatic and asymptomatic cases), vaccination price, and vaccine efficacy on the cost-effectiveness of vaccinating children ≤ 1 year of age against dengue infection. Vaccinating was highly cost-effective (ICER < 4,289) for all scenarios up to a $60 vaccination price point, and dominated (i.e., was less costly and more effective than) not vaccinating in most scenarios at efficacies of at least 75% when the total cost of the 3-dose vaccine was $1.50 or less. Vaccination remained cost-effective (4,289 > ICER < 12,868) through vaccination costs of $200, and remained cost-effective at vaccination price points of $400 if the vaccine efficacy was at least 75% and infection incidence was ≥ 9%. Under conditions where the vaccination cost was $500, vaccinating continued to be cost-effective if the vaccine efficacy was greater than or equal to 85% and incidence rates were ≥ 9%. Vaccination was not cost-effective when infection incidence rates were 9% or less, vaccination cost was $300 or greater, and efficacy was no greater than 50%. When the vaccination cost was $700, a vaccine was only cost-effective when the efficacy was 85% or greater and when infection risks were at least 9%. Vaccinating even proved to be cost-effective with a vaccination price point of $800 when vaccine efficacy was 95% and infection risk was 15%.

### Sensitivity analyses.

Varying the likelihood of minor and major side effects of vaccination, child to adult DHF ratios and of treatment-seeking behaviors did not alter our results in any significant manner.

Varying the probability that primary and secondary symptomatic infection will result in DHF (respectively, 25% and 89%; 15% and 75%; 10% and 50%) only slightly changed our results. In a few scenarios vaccination went from being highly cost-effective to only cost-effective. For example, at baseline risks for development of DHF, a $100 and 50% efficacious vaccine was highly cost-effective (ICER = 3,416) when the annual infection risk was 15%, but only cost-effective for annual infection risks of 5% and 9%. However, changing the risks of developing DHF in primary and secondary symptomatic dengue infections to 10% and 50%, respectively, resulted in ICER values that were only cost-effective for all infection risks (ICER range: 4,946–9,363). A $60 and 50% efficacious vaccine was highly cost-effective at all infection risks for baseline risks for DHF development (ICER range: 2,298–3,881), but with risks for development of DHF of 10% and 50% in primary and secondary symptomatic infections, was cost-effective at an infection rate of 5% while remaining highly cost-effective at infection rates of 9% and 15%.

Decreasing the asymptomatic rate in secondary infections increased the cost-effectiveness of the vaccine. For example, when the asymptomatic rate in secondary infections was decreased from 84% (baseline) to 75%, a $100 vaccine that was 50% efficacious went from being predominantly cost-effective (ICER range: 3,416–6,556) to highly cost-effective (ICER range: 2,581–3,848). At this lower asymptomatic rate the vaccine remained cost-effective up to a $300 vaccination price. Conversely, increasing the asymptomatic rate up to 90% decreased the vaccine's cost-effectiveness somewhat, but only shifted the cost-effectiveness thresholds slightly. For example, at an asymptomatic rate of 90% and a vaccine efficacy rate of 50%, vaccination was cost-effective at a vaccination cost of ≤ $200 (Range: ICER 21,160–11,697). In contrast, at the baseline asymptomatic rate of 84%, vaccination was cost-effective at an increased cost of ≤ $300 (ICER Range: 11,495–19,505).

### Number of dengue and DHF/DSS episodes avoided by vaccination.

Table 2 shows how the number of DHF/DSS episodes per 1,000 vaccinated individuals and total dengue episodes (symptomatic and asymptomatic dengue fever and DHF/DSS) averted over their lifetimes varied by vaccine efficacy and cost, when the infection risk was a baseline 9%. When vaccine efficacy was lowered to 50%, vaccinating 1,000 individuals averted an average of 422.42 episodes of dengue and 39 episodes of DHF/DSS. Vaccinating 1,000 children with a 95% efficacious vaccine prevented an average of 854.67 dengue episodes and 91 DHF/DSS episodes over their lifetimes. Increasing vaccine efficacy from 50–95% more than doubled (average 39 to 91 per 1,000) the number of DHF episodes avoided, and almost doubled (from average 422 to 855 per 1,000) the number of total dengue episodes avoided.

### Cost per averted dengue and DHF/DSS episode.

Table 2 shows how the cost needed to avoid a dengue or DHF/DSS episode increased with increasing vaccine cost and decreasing vaccine efficacy. When the total vaccination cost was ≤ $1.50, vaccination was cost saving (i.e., administering vaccine actually saved money) with a vaccine efficacy of 75% or greater. The cost per averted dengue episode fell below $100 in situations when the total vaccination cost was ≤ $60 and vaccine efficacy was ≥ 75%. The cost to avert a DHF/DSS episode was ~10 times higher than the cost to avert a dengue episode.

## Discussion

This is an important time for an economic evaluation of a dengue vaccine. Although the analysis by Shepard and others provided valuable results, a substantial amount of information has emerged since its publication in 2004. There is now much more extensive vaccine safety, efficacy, and other information (e.g., dose requirements) from phase II trials. With several vaccine candidates now in clinical trials, many stakeholders must begin considering important economic questions about the vaccine: for manufacturers, marketing, pricing, and distribution strategies; for potential purchasers, pricing (and price negotiations), and distribution as well; for scientists, deciding on appropriate efficacy and other vaccine characteristic targets; and for policy makers and clinicians, choosing target populations and understanding risk-benefit profiles. Previous experiences with other vaccines (e.g., LYMERix, FluMist, and rotavirus) have highlighted the importance of thoroughly examining these questions early enough so that appropriate changes and contingency plans can be made.^16^

Our study suggests that the vaccine would be cost-effective for a wide range of efficacies (i.e., as low as 50%) and costs (as high as $800 with an efficacy of 95% and infection risk of 15%). Because vaccine efficacy promises to exceed 50%,^13^ our study suggests that the vaccine may remain cost-effective even in the face of possible waning immunity over time or suboptimal compliance (i.e., patients not getting the full complement of a multiple dose vaccine).^40^ Higher price points may discourage purchasers^16^ but also could encourage more manufacturers to develop the vaccine.^41^ Because our analyses show that the vaccine is cost-effective even for those with lower dengue risk, they support vaccination of most of the Thai population, even if the vaccine itself and other control measures such as vector control were to lower risk of disease. The model does not consider the indirect effects of vaccine, which would include herd immunity. Immunizing vaccines would lower the risk of infection that all individuals in a population would experience. Because this would reduce the risk that vaccinees would experience, it would potentially reduce the cost-effectiveness of vaccination. On the other hand, our present methods do not incorporate the societal benefits of indirect protection that all individuals, vaccinated or not, would gain through vaccine use. We would expect these indirect effects, if included, to increase the cost-effectiveness of vaccine. Future work will consider this benefit. At the same time, by profiling how cost-effectiveness varies with dengue risk, our study may help policy makers identify key target populations for initial immunization. In Thailand for example, assuming vaccination coverage equivalent to that of other 3-dose early childhood vaccines (e.g., DTP, Hep B, Polio) could be achieved, vaccinating the < 1 year old target population was calculated to cost ~$1,436,190 and $14,361,900 annually for a $1.50 and $15 cost of vaccination, respectively (based on 2008 birth cohort estimates).^42^,^43^

## Limitations

All models make simplifying assumptions and cannot represent all possible outcomes of dengue infection, dengue treatment, or possible vaccine side effects. Our analysis assumed complete compliance and did not evaluate vaccine boosters. The model does not consider the indirect effects of vaccine including herd immunity. Future studies may look at the impact of vaccinating different proportions of the populations on vaccinated and non-vaccinated individual's risk of dengue transmission and infection. Achieving adequate levels of herd immunity may reduce infection and disease risk in non-vaccinated members of the population. There is also the possibility that some individuals may be at increased risk for disease. Because of limited specific data of adult primary and secondary dengue infections in Thailand, we extrapolated a ratio from a study in Cuba where recent outbreaks and prospective investigations have allowed for extensive data collection on dengue infections and past immunity in adults and children. Although model assumptions and data inputs were drawn from extensive review of the literature, the sources may vary in quality and model parameters may not hold under all conditions. Finally, although we used widely adopted WHO promulgated thresholds, some countries may have different thresholds for considering a vaccine cost-effective. Currency fluctuations would affect both the model outcomes and the thresholds and therefore should not affect our results. However, large changes in the costs of healthcare resources could make a difference.

## Conclusions

As vaccine candidates get closer to licensure, now is the ideal time to further examine the economic value of a dengue vaccine. Our results suggest that a dengue vaccine could be of considerable economic value even at fairly high price points and low vaccine efficacy. In fact, in some cases, vaccination could provide net cost savings. This study may provide key stakeholders, such as policy makers, scientists, manufacturers, purchasers, and clinicians, with benchmarks to assist their decision making.

## Figures and Tables

**Table 1:** Model Inputs

Variable	Mean (SD)	Distribution type	Ref
**Probability (%)**			
Asymptomatic dengue			
Primary infection	91%		9
Secondary infection	84%		9
DHF*			
Primary infection			
Children	25%†	–	9
Adults	7.2%	–	9,21
Secondary infection			
Children	89%‡	–	9
Adults	25.7%	–	9,21
Death from DF	0.0027%	–	22
Death from DHF	0.155% (0.049%)	Beta	20,22
Vaccine major side effect (YEL-AVD)	Range: 0.0012–3%	Uniform	13,23
Vaccine minor side effect	Range: 10–95%	Uniform	13,24,25
**Cost (2010 USD)**			
Clinic visit	$11.09	–	42
Hospital visit			
DF	$34.74	–	26
DHF	$42.71	–	26
Vaccine minor side effect	$0.31	–	§
Disability weight			
DF	0.197	–	27
DHF	0.555	–	27
**Time**			
School days missed (children)			
DF	4.2	–	28
DHF	5.6	–	28
Work days missed (adults)			
DF	6.6	–	28
DHF	9.9	–	28

YEL-AVD = yellow fever vaccine-associated viscerotropic disease; USD = United States dollar.

* Rates are of symptomatic dengue cases that manifest into dengue hemorrhagic fever (DHF) vs. dengue fever (DF).

† Of primary infections in schoolchildren, ~8.5% were symptomatic, 25% of those developed DHF; i.e., the total percentage of primarily infected students with DHF was 8.5% × 25% = 2.1%.

‡ Reported that of secondary infections in schoolchildren, ~16% were symptomatic and of those symptomatic cases, 89% developed DHF; i.e., 14% (16% × 89%) of secondary infections would lead to DHF.

§ Laosiritaworn Y, personal communication.

**Table 2:** Lifetime health and economic outcomes of dengue vaccination per 1,000 vaccinated*

Vaccine efficacy	Number of averted dengue episodes†	Cost per averted dengue case‡ USD (THB)	Number of averted DHF/DSS episodes†	Cost per averted DHF/DSS case‡ USD (THB)
Vaccination cost $1.50				
50%	423	3.23 (101)	39	34.87 (1,087)
75%	658	−0.23 (−7)	66	−2.29 (−71)
85%	756	−1.02 (−32)	78	−9.89 (−308)
95%	855	−1.74 (−54)	91	−16.42 (−512)
Vaccination cost $15				
50%	422	35.25 (1,099)	39	380.81 (11,867)
75%	659	20.27 (632)	66	203.78 (6,350)
85%	758	16.79 (523)	77	164.32 (5,121)
95%	856	14.03 (437)	91	131.57 (4,100)
Vaccination cost $30				
50%	422	70.86 (2,208)	39	757.74 (23,614)
75%	657	43.15 (1,345)	65	433.14 (13,498)
85%	755	36.68 (1,143)	78	357.43 (11,139)
95%	854	31.56 (984)	91	295.07 (9,195)
Vaccination cost $60				
50%	423	141.62 (4,413)	39	1,526.70 (47,577)
75%	658	88.74 (2,765)	65	891.26 (27,775)
85%	746	76.32 (2,378)	78	742.69 (23,145)
95%	855	66.67 (2,078)	91	626.55 (608,471)
Vaccination cost $100				
50%	422	236.54 (7,371)	39	2,545.94 (79,340)
75%	657	149.69 (4,665)	65	1,509.62 (47,045)
85%	757	129.11 (4,023)	78	1,256.01 (39,141)
95%	854	91.36 (2,847)	91	1,061.80 (33,089)
Vaccination cost $200				
50%	422	473.92 (14,769)	39	5,113.14 (159,342)
75%	659	300.90 (9,377)	66	3,015.89 (93,985)
85%	756	261.36 (8,145)	78	2,545.34 (79,321)
95%	854	230.58 (7,186)	91	2,159.17 (67,287)
Vaccination cost $300				
50%	423	709.13 (22,099)	39	7,628.91 (237,741)
75%	659	452.65 (14,106)	66	4,521.93 (140,918)
85%	756	393.93 (12,276)	78	3,813.18 (118,831)
95%	855	347.53 (10,830)	91	3,265.71 (101,770)
Vaccination cost $400				
50%	423	945.24 (29,457)	39	10,145.75 (316,174)
75%	658	605.57 (18,871)	66	6,028.47 (187,866)
85%	757	525.52 (16,377)	78	5,088.88 (158,586)
95%	853	465.15 (14,496)	91	4,377.52 (136,418)
Vaccination cost $500				
50%	422	1,183.99 (36,897)	39	12,688.70 (395,421)
75%	658	756.97 (23,590)	66	7,556.47 (235,484)
85%	755	659.49 (20,552)	77	6,438.22 (200,636)
95%	855	581.44 (18,120)	91	5,445.09 (169,686)
Vaccination cost $600				
50%	423	1,417.97 (44,188)	39	15,326.05 (477,609)
75%	659	907.82 (28,291)	65	9,163.16 (285,553)
85%	757	790.01 (24,619)	78	7,646.04 (238,275)
95%	856	697.72 (21,743)	91	6,530.53 (203,512)
Vaccination cost $700				
50%	422	1,657.67 (51,658)	39	17,783.49 (554,190)
75%	659	1,059.05 (33,003)	66	10,574.00 (329,520)
85%	756	922.85 (28,759)	78	8,977.13 (279,756)
95%	854	815.86 (25,425)	91	7,689.85 (239,640)
Vaccination cost $800				
50%	422	1,894.55 (59,040)	39	20,291.23 (632,340)
75%	658	1,213.99 (37,832)	65	12,230.44 (381,140)
85%	756	1,055.81 (32,902)	78	10,198.75 (317,826)
95%	855	932.68 (29,065)	91	8805.56 (274,409)

* USD = United States dollar; THB = Thai Baht; DHF = dengue hemorrhagic fever; DSS = dengue shock syndrome.

† Per 1,000 individuals vaccinated.

‡ Negative cost values indicate cost savings.
